# 505 Toxic Epidermal Necrolysis and Stevens Johnson Syndrome: More Than Just Skin Deep

**DOI:** 10.1093/jbcr/iraf019.134

**Published:** 2025-04-01

**Authors:** Crystal Dye, Matthew Bozeman, Victoria Hammond, Will Risinger

**Affiliations:** University of Louisville Burn Center; University of Louisville; University of Louisville; University of Louisville

## Abstract

**Introduction:**

There is increasing literature about the significant psychological morbidity of patients’ with SJS/TEN. Conditions such as anxiety, depression, and post-traumatic stress (PTSD) are some of the more common pathologies associated with these injuries. The hypothesis for this study is, in addition to psychiatric disorders, patients recovered from SJS/TEN suffer disabilities in multiple facets of daily life even after healing of their skin disorder.

**Methods:**

Patients with the diagnosis of SJS/TEN admitted from 2021-2024 were identified via retrospective chart review. Their demographics, hospital outcomes, length of stay, and potential causes were obtained from the electronic medical record. Depression and PTSD screenings were performed at follow up visits (GAD-7, PHQ-9, PTSD). Patients were then interviewed in a non-clinical setting to describe the nature of their life’s changes as a direct result of their TEN/SJS sequela after leaving the hospital. A descriptive analysis was then used based on the responses from each patient.

**Results:**

We identified 34 patients via chart review during the study period. The patients had an average surface area affected of 33% total body surface area (TBSA). Average initial length of stay was 13.84 days. 85% had at least one additional extra dermal organ system involved in their disease. Average total time of screening for mental health disorders was 3.65 years. Anxiety, depression and PTSD scores were 5.8, 7.8 and 1.7 at final screening. Average time from disease onset to interview was 4.65 years. Patients reported a variety of ongoing life alterations: chronic medical conditions, dynamic shifts in their households and interpersonal relationships, abrupt and/or continued financial insecurity, limited access to appropriate resources, and complete relearning in managing their activities of daily living.

**Conclusions:**

TEN/SJS is not only an acute condition that ends when skin healing is complete. Deeper emotional and financial injuries persist. Chronic mild depression and anxiety exist even years later. Sequela of physical changes results in significant alterations in emotional, familial, and financial stability. Provider understanding of these non-standardized, long-term effects is an aspect of care that is largely missing and worsens negative outcomes and reintegration into society.

**Applicability of Research to Practice:**

Greater understanding of the long-term outcomes after hospitalization and recovery from acute SJS/TEN is an area of need. Patients will suffer predictable psychosocial and physical symptoms, and verified burn centers should develop and lead multidisciplinary teams to assist in this area of chronic care.

**Funding for the Study:**

N/A

